# Knock Out of S1P3 Receptor Signaling Attenuates Inflammation and Fibrosis in Bleomycin-Induced Lung Injury Mice Model

**DOI:** 10.1371/journal.pone.0106792

**Published:** 2014-09-08

**Authors:** Ken Murakami, Masataka Kohno, Masatoshi Kadoya, Hidetake Nagahara, Wataru Fujii, Takahiro Seno, Aihiro Yamamoto, Ryo Oda, Hiroyoshi Fujiwara, Toshikazu Kubo, Satoshi Morita, Hiroshi Nakada, Timothy Hla, Yutaka Kawahito

**Affiliations:** 1 Inflammation and Immunology, Graduate school of Medical Science, Kyoto Prefectural University of Medicine, Kyoto, Japan; 2 Department of Rheumatic Diseases and Joint Function, Kyoto Prefectural University of Medicine, Kyoto, Japan; 3 Department of Orthopedics, Graduate School of Medical Science, Kyoto Prefectural University of Medicine, Kyoto, Japan; 4 Department of Biomedical Statistics and Bioinformatics, Kyoto University Graduate School of Medicine, Kyoto, Japan; 5 Department of Molecular Biosciences, Faculty of Life Sciences, Kyoto Sangyo University, Kyoto, Japan; 6 Department of Pathology and Laboratory Medicine, Weill Cornell Medical College, New York, New York, United States of America; University of Rochester Medical Center, United States of America

## Abstract

Sphingosine-1-phosphate (S1P) is a bioactive sphingolipid metabolite involved in many critical cellular processes, including proliferation, migration, and angiogenesis, through interaction with a family of five G protein–coupled receptors (S1P1–5). Some reports have implicated S1P as an important inflammatory mediator of the pathogenesis of airway inflammation, but the role of S1P3 in the pathogenesis of lung diseases is not completely understood. We used S1P3-deficient (knockout (KO)) mice to clarify the role of S1P3 receptor signaling in the pathogenesis of pulmonary inflammation and fibrosis using a bleomycin-induced model of lung injury. On the seventh day after bleomycin administration, S1P3 KO mice exhibited significantly less body weight loss and pulmonary inflammation than wild-type (WT) mice. On the 28th day, there was less pulmonary fibrosis in S1P3 KO mice than in WT mice. S1P3 KO mice demonstrated a 56% reduction in total cell count in bronchoalveolar lavage fluid (BALF) collected on the seventh day compared with WT mice; however, the differential white blood cell profiles were similar. BALF analysis on the seventh day showed that connective tissue growth factor (CTGF) levels were significantly decreased in S1P3 KO mice compared with WT mice, although no differences were observed in monocyte chemotactic protein-1 (MCP-1) or transforming growth factor β1 (TGF-β1) levels. Finally, S1P levels in BALF collected on the 7th day after treatment were not significantly different between WT and S1P3 KO mice. Our results indicate that S1P3 receptor signaling plays an important role in pulmonary inflammation and fibrosis and that this signaling occurs via CTGF expression. This suggests that this pathway might be a therapeutic target for pulmonary fibrosis.

## Introduction

Pulmonary fibrosis is a devastating disorder that is resistant to treatment [1]. Initial injury to the lung causes the recruitment of inflammatory cells, release of cytokines, and eventual increase in fibroblast activity, leading to parenchymal remodeling and, finally, fibrosis [2]. Although various cytokines and growth factors are involved in these responses, transforming growth factor (TGF-β) is known to play the most essential role in the pathogenesis of lung fibrosis [3]. Sphingosine-1-phosphate (S1P) is a bioactive sphingolipid metabolite involved in many critical cellular processes, including proliferation, differentiation, migration, and angiogenesis, through interaction with a family of five G protein–coupled receptors (S1P1–5) [4]. In dendritic cells, S1P3 is reported to play a critical role in regulating inflammation in sepsis syndrome via cross-talk with PAR1 [5]. S1P3 also mediates the chemotactic effects of S1P in macrophages *in vitro* and *in vivo*, and plays a causal role in atherosclerosis by promoting inflammatory monocyte/macrophage recruitment [6]. With regard to S1P receptor profiles in neutrophils, S1P1, S1P4, and S1P5 are reported to be expressed on neutrophils in both patients with pneumonia and healthy subjects, while S1P3 receptor expression is observed only on neutrophils from patients with pneumonia [7]. S1P3 also mediates cardiac fibrosis [8] and cholestasis-induced liver fibrosis [9] and thus some current studies suggest a relationship between S1P3 receptor activity and lung inflammation and fibrosis; however, the role of S1P3 in the pathogenesis of lung diseases is still poorly understood. In this study, we analyzed an *in vivo* model of bleomycin-induced pulmonary injury in S1P3 KO mice to clarify the role of S1P3 receptor signaling.

## Methods

### Animals

Animal care and experimental procedures were approved by the Kyoto Sangyo University Committee on Animal Welfare (approval number 2010-52). S1P3^−/−^ (knockout (KO)) mice were generated on a mixed C57BL/6-129/Sv background as described previously [10]. They were backcrossed more than six times to the control C57BL/6J strain. KO littermate mice were bred in the Animal Resource Facility at Kyoto Sangyo University under specific pathogen-free conditions. S1P3^+/+^ (wild-type, (WT)) C57BL/6J strain mice were purchased from Shimizu Laboratory Supplies Co. (Kyoto, Japan). Ltd. The S1P3 genotypes were determined by PCR analyses of genomic DNA isolated from tail biopsy specimens [10]. Mice were sacrificed by intraperitoneal administration of an excessive dose of pentobarbital (120–150 mg/kg), and mice were euthanized when their weights fell below 80% of baseline during an experimental period.

### Bleomycin mouse model

Seven- to ten-week-old mice were anesthetized by ether inhalation and 30 µL of bleomycin hydrochloride (Nippon Kayaku Co, Tokyo, Japan) solution containing 2.15 U/kg of bleomycin dissolved in sterile saline [11] was delivered by direct injection into the trachea using a 0.9 mm feeding needle (KN-348, Natsume Seisakusho Co, Tokyo, Japan). Control group mice received the same volume of sterile saline. The body weight of the mice was measured twice a week for 28 days after the intratracheal administration of bleomycin.

### Histology

Histological analysis was performed on formalin-fixed, paraffin-embedded lung tissue sections stained with H&E or by Masson's trichrome method. The sections were taken at 7 or 28 days after intratracheal administration of bleomycin or saline. The degree of fibrosis was quantified using the Ashcroft scoring method [12] by observers who were blinded as to whether the samples were from WT or KO mice.

### Analysis of bronchoalveolar lavage fluid

BALF analyses were performed on the seventh day after bleomycin administration. Immediately after the mice were sacrificed, the lungs and trachea were extracted en bloc, and a 20-gauge intravenous catheter was inserted into the trachea. A total of 800 µL of PBS was instilled three times and withdrawn from the lungs via an intratracheal cannula. More than 90% of the fluid was recovered as bronchoalveolar lavage fluid (BALF), which was then centrifuged at 1,000 rpm for 5 minutes at 4°C. The supernatants were collected and stored at −80°C for ELISA. For the analysis of total and differential white blood cell counts, after removing the supernatant, the resultant pellet was washed with PBS and resuspended in 700 µL of PBS. A 200 µL aliquot of the 700 µL BALF solution was diluted with 400 µL of Turk's solution and the total number of BALF cells was counted using a Fuchs-Rosenthal hemocytometer (ERMA Inc, Tokyo, Japan). The rest of the BALF solution was placed in a cytospin (Cytospin 2; Shandon Inc, Pittsburgh, PA, USA), centrifuged at 700 rpm for 10 minutes and stained with Diff-Quick (Sysmex, Kobe, Japan) to obtain the differential white blood cell count. At least 200 cells per slide were evaluated on the basis of morphological criteria using a light microscope. The concentrations of TGF-β1, monocyte chemotactic protein-1 (MCP-1), connective tissue growth factor (CTGF), and S1P were measured with ELISA kits according to the manufacturer's instructions. ELISA kits for TGF-β1 and MCP-1 were provided by eBioscience (San Diego, CA, USA), for CTGF by Uscn Life Science (Houston, TX, USA) and for S1P by Echelon Biosciences Inc. (Salt Lake City, UT, USA). Collagen content was estimated by collagen assay using a Sircol collagen assay kit (Biocolor, Northern Ireland, U.K.).

### Statistical analysis

The data are presented as the mean ± SD and all sample sizes were ≥5. All values except S1P levels were analyzed by Welch's t-test. Differences in S1P levels in BALF between WT and S1P3 KO mice were analyzed by Mann-Whitney U test. Differences were considered significant at the P<0.05 level.

## Results

### Changes in body weight

**Figure 1:**
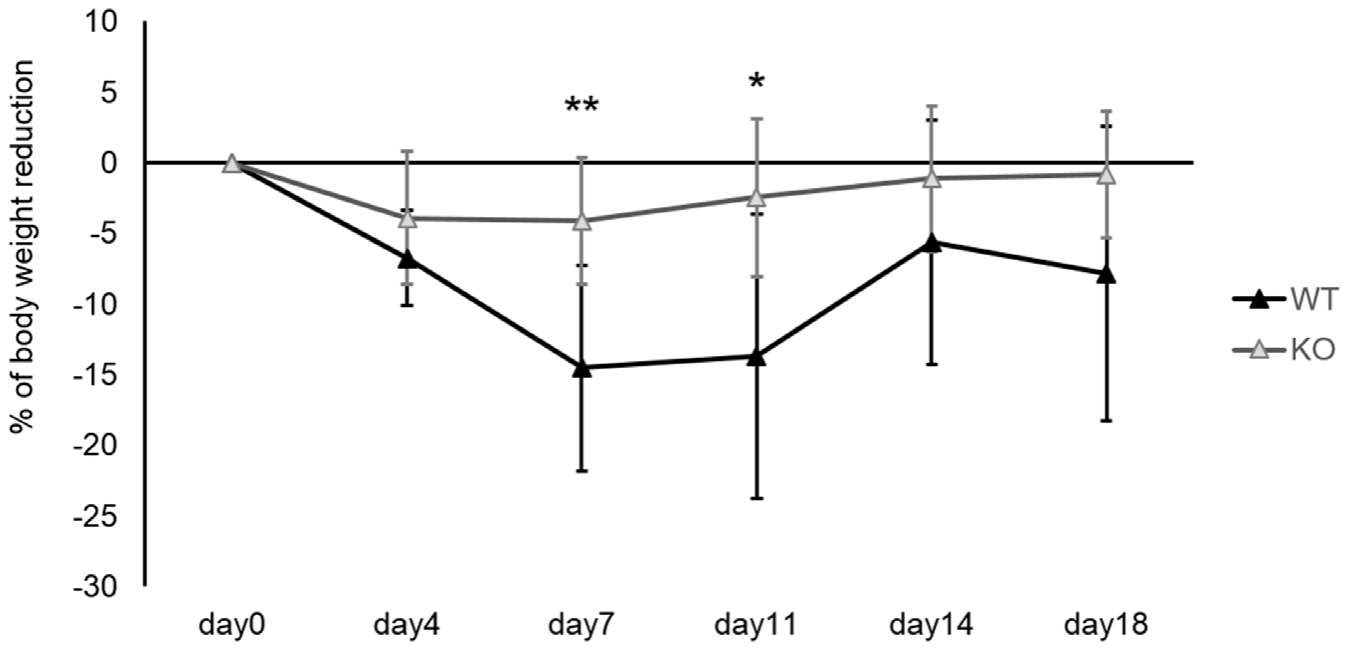
Changes in body weight in mice after administration of bleomycin. Time course of changes in body weight in wild-type (WT) (*n* = *9*) and in S1P3 knockout (KO) (*n* = *8*) mice after administration of bleomycin. S1P3 KO mice exhibited decreased body weight loss compared to WT mice. The weight of the WT mice gradually decreased, reaching their lowest point on the seventh day after treatment and increasing afterwards. Values are presented as the mean ± SD (*p<0.05, **p<0.01).

To determine the biological significance of S1P3 deficiency after acute lung injury, we tracked changes in body weight after exposure to bleomycin. The average body weight at baseline did not differ significantly between WT (n = 9) and S1P3 KO mice (n = 8). After treatment with bleomycin, body weights in both groups decreased, but weight loss was significantly reduced in S1P3 KO mice (Figure 1A). The body weights of WT mice decreased to approximately 85% of baseline by 7 days after treatment and gradually recovered afterwards, whereas the body weights of S1P3 KO mice decreased to approximately 95% of baseline. Mice whose weights were less than 80% of baseline were euthanized. Four WT mice were euthanized on the seventh or eleventh day after treatment; however, the body weights of all KO mice were above 80% of the baseline during the observation period.

To determine the survival rate after administration of bleomycin, data from four independent experiments were combined. The survival rate of WT mice decreased to 61.8% (21/34) on the eleventh day and that of S1P3 KO mice to 87.1% (27/31). The survival rate of S1P3 KO mice after administration of bleomycin was significantly higher than that of WT mice (p = 0.039; the data were analyzed by log-rank test) (Figure S1).

### Histological evaluation

**Figure 2:**
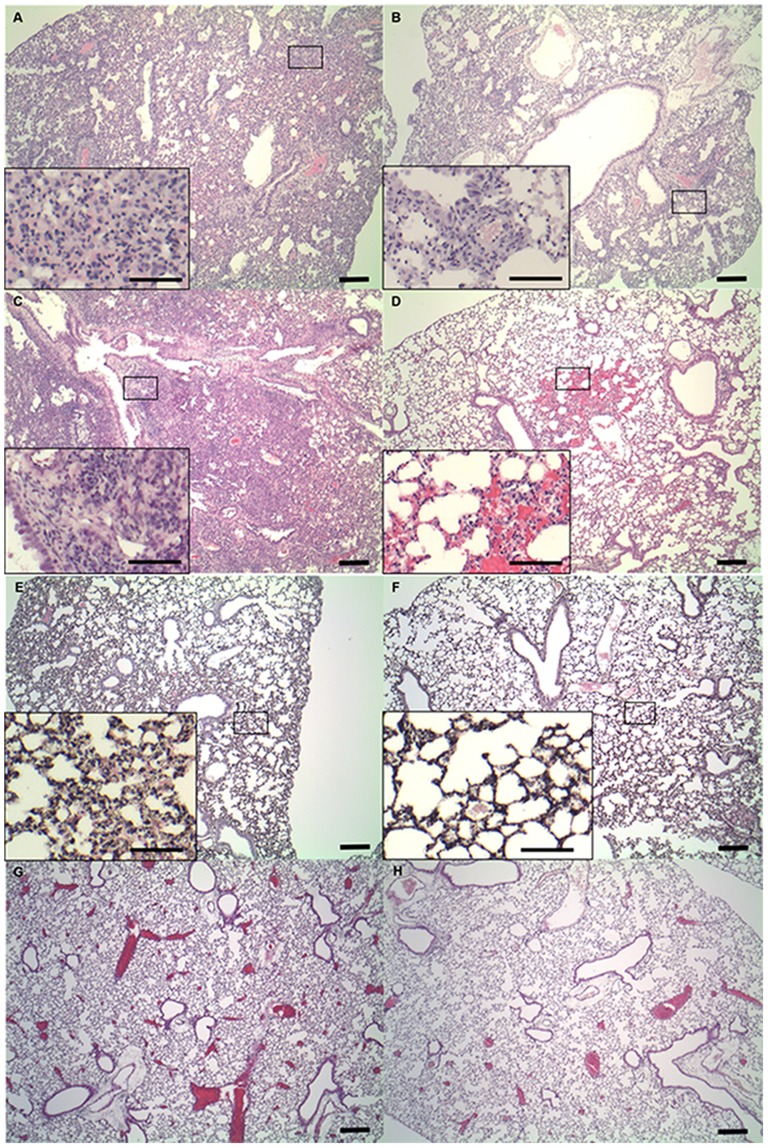
Histopathological findings of pulmonary inflammation. Representative results of H&E staining of lung tissue from mice in the bleomycin-induced lung injury model (A, B, C and D). H&E staining of lung tissue from (A) wild-type (WT) and (B) knockout (KO) mice on the seventh day after intratracheal administration of bleomycin. H&E staining of lung tissue from (C) WT and (D) KO mice on the 28th day after intratracheal administration of bleomycin. All scale bars  = 100 µm. The window shows an area of increased magnification revealing inflammatory cell infiltration. Representative results of H&E staining of lung tissue from mice in the saline-treated control group (E, F, G and H). H&E staining of lung tissue from (E) WT and (F) KO mice on the seventh day after intratracheal administration of saline. H&E staining of lung tissue from (G) WT and (H) KO mice on the 28th day after intratracheal administration of saline. All scale bars  = 100 µm. The window shows an area of increased magnification revealing hyperplasia of the alveolar/bronchiolar epithelium.

Lung tissues from WT and S1P3 KO mice on the seventh and 28th day after treatment with bleomycin or saline were stained with H&E or Masson's trichrome staining to evaluate histological changes during the acute and chronic phases of lung injury. On the seventh day after treatment, inflammation was apparent with slight progression of fibrosis in lung tissues of WT mice, which showed infiltration of inflammatory cells, hyperplasia of the alveolar/bronchiolar epithelium, and interstitial collagen deposition in affected lesions. Lung tissues from S1P3 KO mice exhibited less inflammation than those from WT mice (Figure 2A, B). On the 28th day after treatment, inflammation and fibrosis were readily apparent in the lung tissues from WT mice, while tissues from S1P3 KO mice exhibited less inflammation and fibrosis (Figure 2C, D). In mice treated with saline, lung tissues exhibited less inflammation than the bleomycin-treated group (Figure 2E–H). On the seventh day after treatment with saline, hyperplasia of the alveolar/bronchiolar epithelium in S1P3 KO mice was slightly less than in WT mice (Figure 2E, F), and no significant differences in the degree of inflammation between WT and KO mice were observed on the 28th day after treatment with saline (Figure 2G, H).

**Figure 3:**
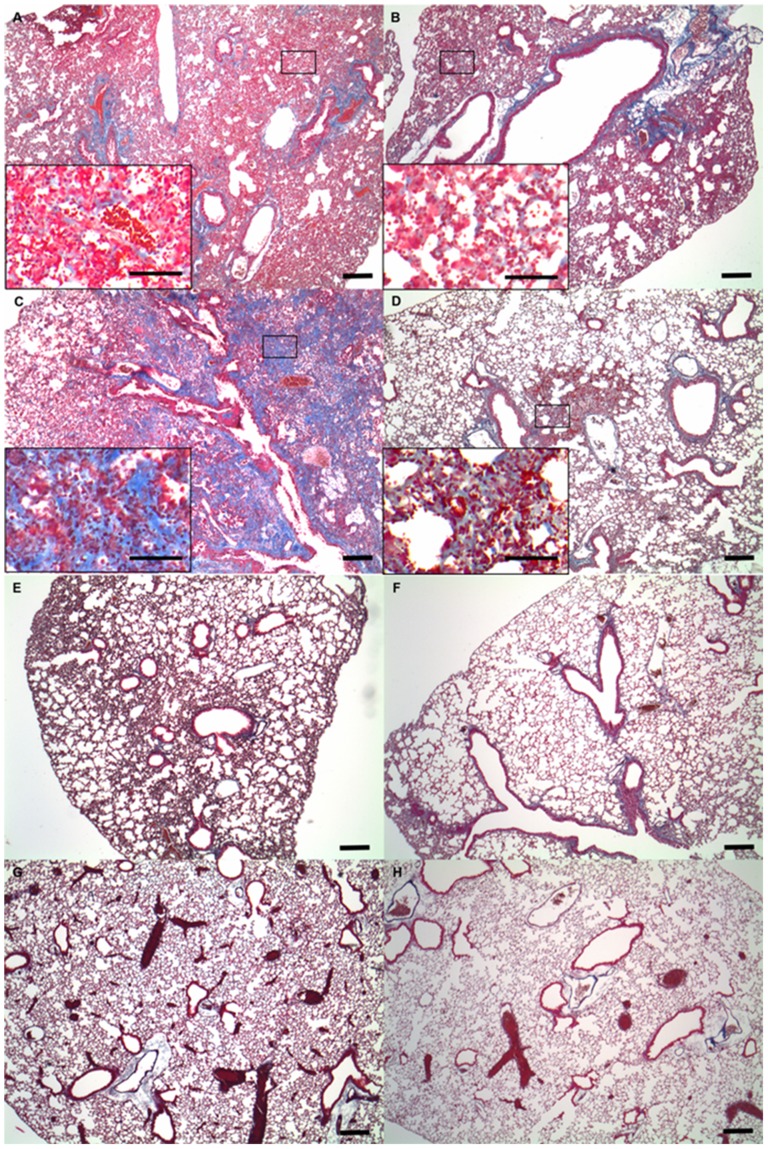
Histopathological findings of fibrosis induced by bleomycin. Representative results of Masson's trichrome staining of lung tissue from mice in the bleomycin-induced lung injury model (A, B, C and D). Masson's trichrome staining of lung tissue from (A) wild-type (WT) and (B) knockout (KO) mice on the seventh day after intratracheal administration of bleomycin. Masson's trichrome staining of lung tissue from (C) WT and (D) KO mice on the 28th day after intratracheal administration of bleomycin. All scale bars  = 100 µm. The window shows an area of increased magnification revealing a part of lung fibrosis. Representative results of Masson's trichrome staining of lung tissue from mice in the saline-treated control group (E, F, G and H). Masson's trichrome staining of lung tissue from (E) WT and (F) KO mice on the seventh day after intratracheal administration of saline. Masson's trichrome staining of lung tissue from (G) WT and (H) KO mice on the 28th day after intratracheal administration of saline.

When fibrosis was examined by Masson's trichrome staining of lung tissues, no significant differences in fibrosis on the seventh day after treatment were detected in either group (Figure 3A, B). However, on the 28th day after treatment with bleomycin, severe fibrosis was evident in lung tissues from WT mice (Figure 3C), while lung tissues from S1P3 KO mice exhibited less fibrosis (Figure 3D). In control mice treated with saline, almost no fibrosis was observed in the lungs on either the seventh or 28th day after treatment (Figure 3E–H).

**Figure 4:**
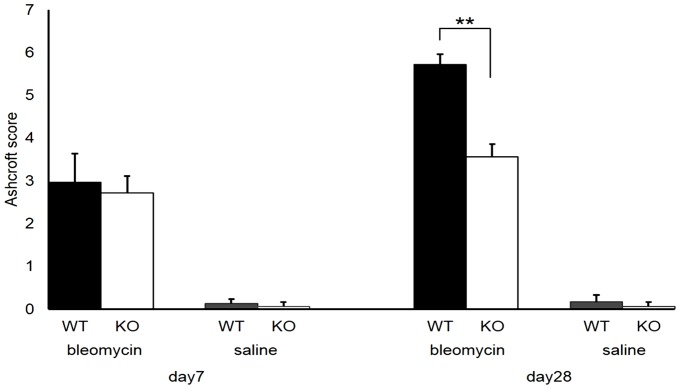
Ashcroft score. Ashcroft score of wild-type (WT) and S1P3 knockout (KO) mice on the seventh and 28th day after treatment with bleomycin or saline. No significant differences were observed between the Ashcroft scores of KO mice (2.72±0.30) and WT mice (2.96±0.23) on the seventh day after treatment with bleomycin. The Ashcroft scores of KO mice (3.56±0.39) were significantly lower than those of WT mice (5.72±0.68, *p* = 0.0006, *n* = 5 in each genotype) on the 28th day. In saline-treated control mice, almost no fibrosis of the lungs was observed on either the seventh day (0.13±0.09 vs. 0.07±0.09 in WT vs. KO mice, respectively; *n* = 3 in each genotype) or the 28th day (0.17±0.17 vs. 0.07±0.09 in WT vs. KO mice, respectively; *n* = 3 in each genotype).

No significant differences were recognized in the Ashcroft score of KO mice (2.72±0.30) and WT mice (2.96±0.23, *p* = 0.12) (Figure 4) on the seventh day after treatment. However, on the 28th day after treatment, the Ashcroft score of S1P3 KO mice (3.56±0.39) was significantly less than that of WT mice (5.72±0.68, *p* = 0.0006) (Figure 4). In saline-treated control mice, little fibrosis of the lungs (Figure 4) was evident on either the seventh or 28th day.

### Analysis of BALF

**Figure 5:**
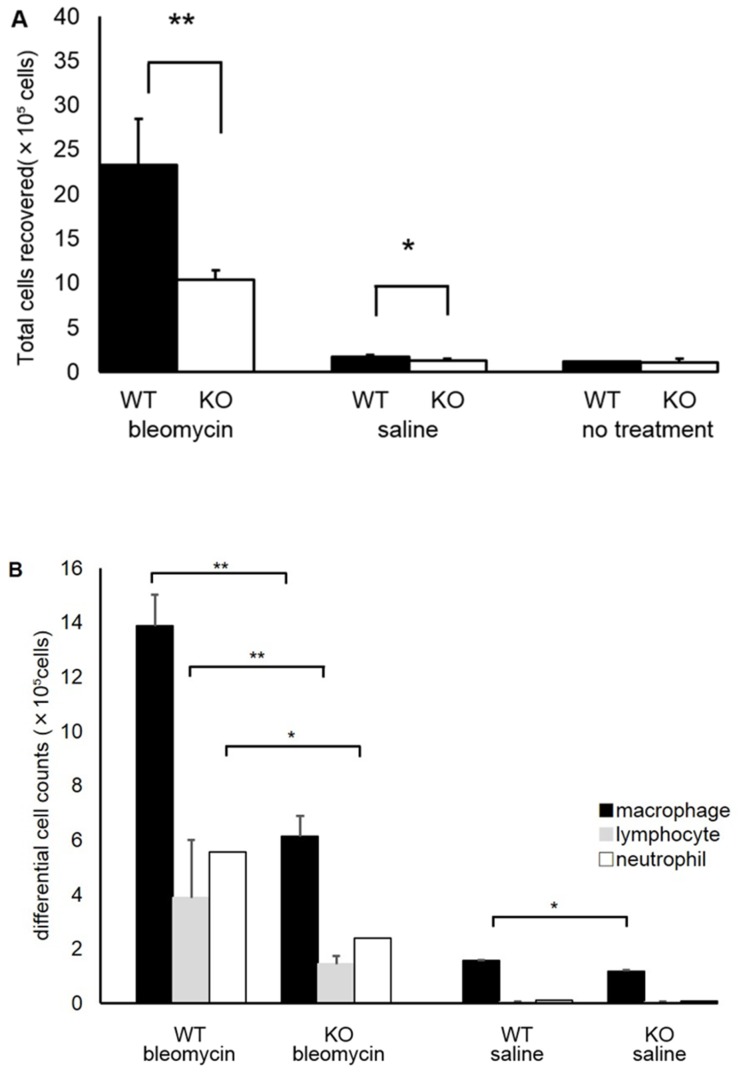
Total and differential white blood cell counts in bronchoalveolar lavage fluid (BALF). A. Total cell count in BALF on the seventh day after intratracheal administration of bleomycin. Total cell counts in BALF collected on the seventh day after treatment were reduced by 56% in S1P3 knockout (KO) mice compared with wild-type (WT) mice (*n* = 5 in each genotype; **p<0.01). In saline-treated control mice, total cell counts in BALF collected on the seventh day after treatment were reduced by 25% in S1P3 KO mice compared with WT mice (n = 3 in each genotype, *p<0.05). Values are presented as the mean ± SD. B. Differential white blood cell counts in BALF collected after intratracheal administration of bleomycin or saline. The differential white blood cell counts from WT and KO mice had similar profiles (*n* = 5 in each genotype). Values are presented as the mean ± SD.

Analysis of BALF collected on the seventh day after treatment revealed a 56% reduction in total cell count in S1P3 KO mice compared with WT mice (*n* = 5, (2.33±0.52) ×10^6^ cells vs. (1.03±0.11) ×10^6^ cells in WT vs. KO mice, respectively; *p* = 0.003) (Figure 5A). However, for the differential cell counts, S1P3 KO and WT mice had similar profiles (WT vs KO; macrophage: (13.87±3.7) ×10^5^ cells (59.3±5.2%) vs (6.13±1.13) ×10^5^ cells (59.1±3.8%), lymphocyte: (3.88±1.13) ×10^5^ cells (16.9±3.8%) vs (1.44±0.76) ×10^5^ cells (17.3±1.1%), neutrophil: (5.56±0.21) ×10^5^ cells (23.8±4.7%) vs (2.41±0.21) ×10^5^ cells (23.6±3.8%), Figure 5B). In saline-treated control mice, total cell counts in BALF from S1P3 KO mice on the seventh day after treatment was decreased by 25% compared to WT mice (*n* = 3; (1.74±0.13) ×10^5^ cells vs. (1.31±0.21) ×10^5^ cells in WT vs. KO, respectively; *p* = 0.02) (Figure 5A). Like bleomycin-treated mice, saline-treated S1P3 KO and WT mice also had similar differential cell profiles on the seventh day (WT vs KO; macrophage: (1.58±0.17) ×10^5^ cells (91.4±4.6%) vs (1.19±0.22) ×10^5^ cells (90.5±4.0%), lymphocyte: (0.05±0.008) ×10^5^ cells (2.8±1.2%)vs (0.05±0.02) ×10^5^ cells (3.6±1.7%), neutrophil: (0.10±0.03) ×10^5^ cells (5.8±3.4%) vs (0.08±0.02) ×10^5^ cells (5.9±0.8%); Figure5B). There were no differences of cell numbers between non-treated WT and KO mice (*n* = 3; WT: (1.12±0.07) ×10^5^ cells vs KO: (1.08±0.42) ×10^5^ cells; *p* = 0.44, Figure5A).

**Figure 6:**
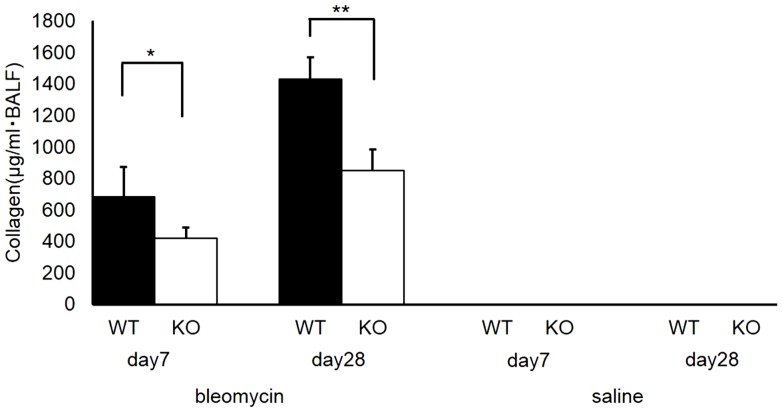
Collagen levels in bronchoalveolar lavage fluid (BALF). Collagen levels in BALF collected on the seventh and 28th day after intratracheal administration of bleomycin or saline. Knockout (KO) mice had significantly less pulmonary fibrosis compared with wild-type (WT) mice on the seventh day (*n* = 5 in each genotype, *p<0.05) and the 28th day (*n* = 5 in each genotype, **p<0.01). In saline-treated control mice, collagen levels were undetectable on both the seventh and 28th days.

S1P3 KO mice had significantly lower collagen levels in BALF compared with WT mice on the seventh day after bleomycin administration (*n* = 5; 683.7±187.0 µg/ml vs. 421.0±67.3 µg/ml in WT vs. KO, respectively; *p* = 0.02) (Figure 6). The difference was more pronounced on the 28th day after bleomycin administration (*n* = 5; 1,430.4±139.7 µg/ml vs. 850.1±131.4 µg/ml in WT vs. KO, respectively; *p* = 0.00015) (Figure 6). In saline-treated control mice, the collagen levels were below the limit of detection in both the acute and chronic phases.

**Figure 7:**
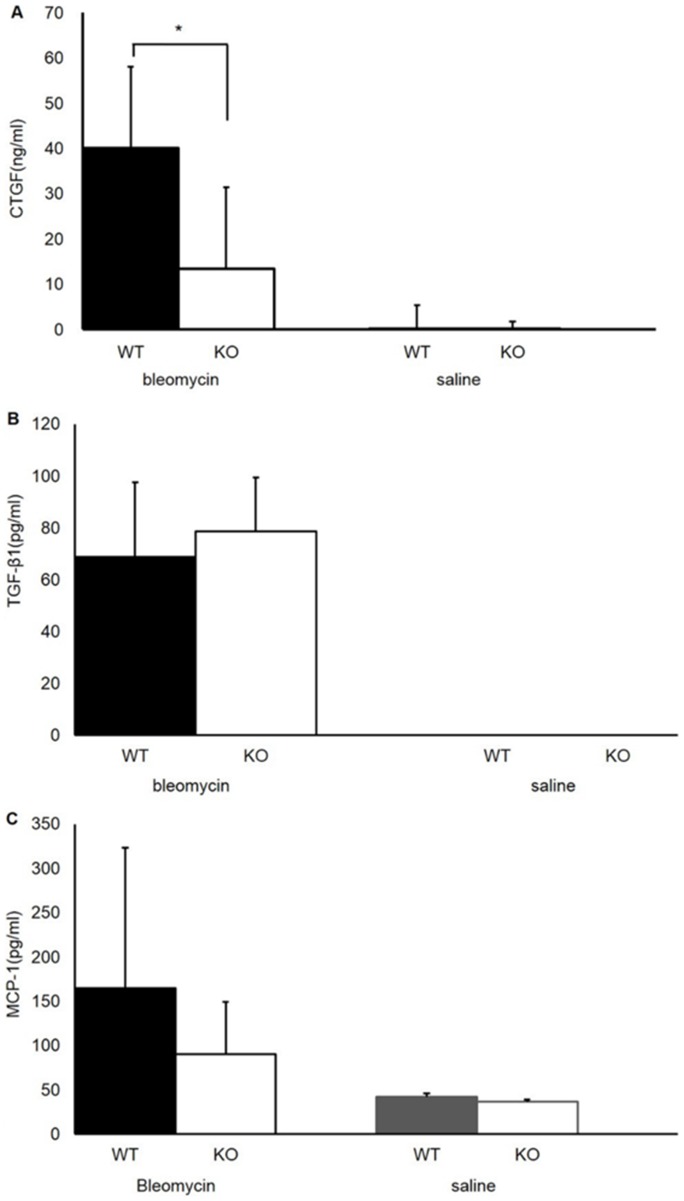
ELISA analyses of bronchoalveolar lavage fluid (BALF). A. CTGF concentrations in BALF collected on the seventh day after intratracheal administration of bleomycin or saline. The CTGF concentrations in BALF after administration of bleomycin were significantly decreased in S1P3 knockout (KO) mice compared with wild-type (WT) mice (*n* = 5 in each genotype, *p<0.05). Values are presented as the mean ± SD. B. TGF-β1 concentrations in BALF collected on the seventh day after intratracheal administration of bleomycin or saline. No significant differences between WT and KO mice were observed in the concentration of TGF-β1 in BALF collected after administration of bleomycin (*n* = 5 in each genotype, *p* = 0.30). Values are presented as the mean ± SD. C. MCP-1 concentrations in BALF on the seventh day after intratracheal administration of bleomycin or saline. No significant differences between WT and KO mice were observed in the concentration of MCP-1 measured in BALF collected after administration of bleomycin (*n* = 5 in each genotype, *p* = 0.23). Values are presented as the mean ± SD.

The CTGF concentration in BALF on the seventh day after intratracheal administration of bleomycin was significantly decreased in S1P3 KO mice compared with WT mice (n = 5; 40.2±18.0 ng/mL vs. 13.5±18.0 ng/mL in WT vs. KO mice, respectively; *p*<0.05) (Figure 7A). However, no significant differences were observed between WT and S1P3 KO mice in the levels of TGF-β1 (n = 5; 68.7±28.9 pg/mL vs. 78.5±20.9 pg/mL in WT vs. KO, respectively; *p* = 0.30) (Figure 7B) or MCP-1 (n = 5; 164.8±158.4 pg/mL vs. 90.1±59.0 pg/ml in WT vs. KO, respectively; *p* = 0.20) (Figure 7C) the seventh day after intratracheal administration of bleomycin. In saline-treated control mice, no significant differences were observed in MCP-1 and CTGF concentrations, and TGF-β1 concentrations were below the limit of detection in both WT (*n* = *3*) and S1P3 KO (*n* = *3*) mice.

**Figure 8:**
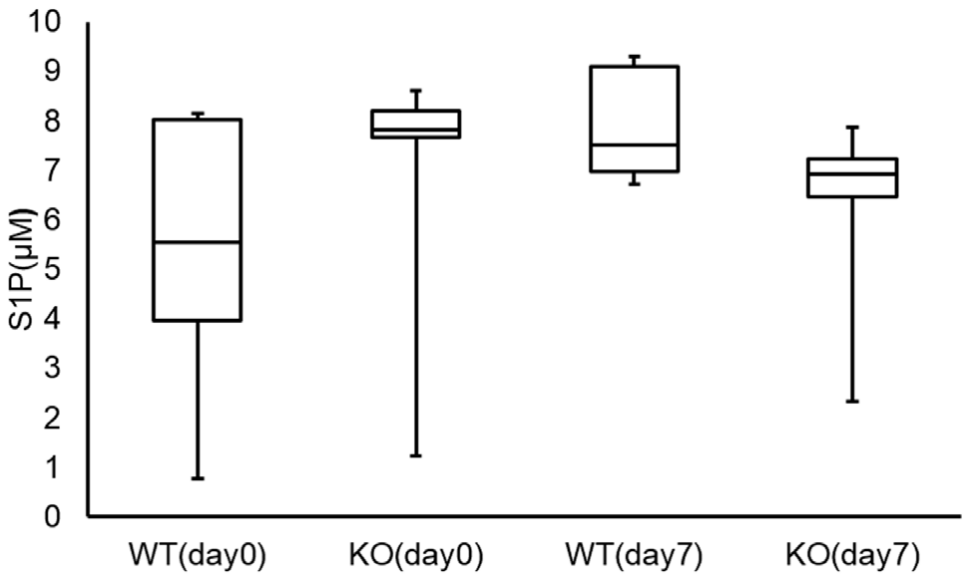
Concentration of S1P in bronchoalveolar lavage fluid (BALF). S1P concentration in BALF collected on the seventh day after intratracheal administration of bleomycin or non-treated control. There were no significant differences in S1P concentration in BALF between wild-type (WT) and knockout (KO) mice at baseline or on the seventh day after bleomycin administration, nor were there significant differences in the S1P concentrations in BALF collected before and after bleomycin administration in WT (baseline vs. seventh day; *p* = 0.14) or KO (baseline vs. seventh day; *p* = 0.50) mice.

Finally, there were no significant differences between the S1P concentrations in BALF from WT and KO mice at baseline or on the seventh day (Figure 8) after bleomycin administration, nor did we detect significant differences in S1P levels in BALF collected before and after bleomycin challenge in WT or S1P3 KO mice.

## Discussion

In this study, we demonstrated that S1P3 deficiency has a significant effect on the pathogenesis of bleomycin-induced pulmonary inflammation and fibrosis. In general, the murine model of bleomycin-induced injury initially showed an acute inflammatory reaction when there was loss of body weight, followed by a fibrotic reaction. In this study, the loss of body weight in S1P3 KO mice was significantly attenuated, suggesting that the inflammatory response to bleomycin was reduced and that S1P3 deficiency was protective against the decline in physical health and feeding induced by bleomycin. During the acute phase of lung injury, H&E staining revealed less histological evidence of inflammation in the lungs of S1P3 KO mice than in those of WT mice, and analysis of BALF collected on the seventh day showed that S1P3 KO mice had a 56% reduction in total cell count compared with WT mice. These results are consistent with the observed changes in body weight.

Sammani et al. showed that compared with control mice, S1P2 KO mice as well as mice with reduced S1P3 expression via silencing S1P3-containing nanocarriers were protected against LPS-induced barrier disruption [13]. Singleton et al. also demonstrated that the activation of S1P3, which is expressed in both the alveolar epithelium and lung vascular endothelium, resulted in robust Rho/Rho kinase-mediated endothelial cell barrier disruption [14], [15]. These reports suggest a pro-inflammatory role for S1P3 in lung injury and are consistent with our results showing that S1P3 KO mice had less bleomycin-induced lung inflammation. In addition, these findings appear to be consistent with the results of our BALF analyses, which showed a reduction in total cell counts, but no differences in differential white blood cell profiles. On the other hand, some reports suggest an anti-inflammatory role for S1P in lung injury. Intravenous administration of S1P significantly decreased pulmonary vascular leakage and inflammation in a murine model of LPS-mediated acute lung injury and canine models of acute lung injury induced by combined intrabronchial endotoxin administration and high tidal volume mechanical ventilation [16], [17]. Shea et al. showed that short-term administration of S1P1 agonists prevented vascular leaks in models of acute lung injury; however, after prolonged exposure to these agents, they acted as functional antagonists of S1P1 and worsened pulmonary vascular leakage after injury [18]. This suggests that the effect of S1P on pulmonary inflammation depends on the concentration of S1P, the time course of the disease, and the expression of the receptor subtypes.

Keul et al. reported that S1P is chemotactic for monocytes/macrophages via the S1P3 receptor, and bone marrow-derived S1P3-deficient macrophages produced less MCP-1 in response to LPS stimulation *in vitro* [6]. In our study, the concentration of MCP-1 was not significantly reduced in KO mice, although the number of macrophages was decreased in BALF. This difference can be explained by the fact that MCP-1 arises from multiple sources in addition to macrophages, such as epithelium and endothelium, and under these conditions, macrophages might not make a significant contribution to MCP-1 levels.

In saline-treated control mice, analysis of BALF collected on the seventh day revealed a 25% reduction in total cell count in S1P3 KO mice compared with WT mice, while there were no differences in cell numbers between non-treated WT and KO mice. It is possible that microaspiration occurred in saline-treated control mice, and that the differences in the total number of cells in BALF occurred due to inflammation caused by aspiration.

Idiopathic pulmonary fibrosis (IPF) is the most common interstitial fibrotic pulmonary disease; however, there is no effective treatment for preventing the development of fibrosis in IPF. Recent studies have implicated S1P, SphK and S1P receptors in human IPF. Huang et al. reported that the expression of SphK1 was increased in lung tissues from patients with IPF and bleomycin-challenged mice. They also showed that knockdown of SphK1 or treatment with an SphK inhibitor attenuated S1P generation and reduced mortality and pulmonary fibrosis in bleomycin-challenged mice [19]. Milara et al. reported that S1P levels in serum and BALF were significantly higher in patients with IPF than in control samples. They also showed that S1P levels in BALF were inversely correlated with lung function, but found no correlation between serum S1P levels and clinical features of the disease. In addition, S1P levels did not correlate with leukocyte or lymphocyte numbers in BALF [20]. Using a mouse model, Gorshkova et al. showed that radiation-induced pulmonary fibrosis is characterized by a marked upregulation of S1P levels in both the lung tissue and in circulation and that this is accompanied by increased lung SphK1 expression and activity [21]. These reports suggest that S1P signaling (SphK1, S1P, and S1P receptors etc.) has a facilitatory effect on pulmonary fibrosis. On the other hand, Mathew et al. reported that mice with targeted deletion of SphK1 (SphK1−/−) or with reduced expression of S1P receptors (S1P1+/−, S1P2−/−, and S1P3−/−) exhibited markedly increased susceptibility to radiation-induced lung injury, and that S1P analogs (S1P1 agonists such as SEW-2871 and the two S1P analogs, FTY720 and (*S*)-FTY720-phosphonate) reduced the degree of radiation-induced lung injury [22]. This suggests that S1P signaling plays a protective role in radiation-induced pulmonary fibrosis.

The standard animal model for induction of experimental pulmonary fibrosis is the intratracheal bleomycin model. This model does not recapitulate the progressive and irreversible characteristics of human IPF; however, histological hallmarks, such as intra-alveolar buds, mural incorporation of collagen and obliteration of the alveolar space, are present in bleomycin-treated animals [23].

Although S1P signaling is implicated in lung fibrosis, its function is not completely understood. It may depend on the pathophysiology or time course of the disease, or it may depend on the concentration of S1P and the expression of receptor subtypes. Our results showed that S1P levels in BALF did not change between baseline and the seventh day after bleomycin challenge, regardless of whether S1P3 was knocked out. Differences between the conditions in the above reports and our study could account for these results, such as variations in SphK activity, different sample sources (total lung tissue, serum or BALF), or different disease time courses. Although previous reports have not examined the expression or distribution of S1P3 in the lungs of IPF patients or bleomycin-induced fibrosis models directly, our results showed that the degree of pulmonary fibrosis and the concentration of CTGF in BALF were dependent on the expression of S1P3, not differences in S1P levels in WT and S1P3 KO mice.

TGF-β is a known profibrotic protein and is considered a key player in the pathogenesis of fibrosis. It is synthesized by different cell types, such as monocytes, lymphocytes, or eosinophils, which are recruited to the site of injury or inflammation. It induces the transformation of fibroblasts into myofibroblasts, which are able to secrete TGF-β and stimulate extracellular matrix (ECM) deposition [24], [25]. CTGF is another important fibrogenic factor. Because CTGF is potently induced by TGF-β, it is considered a downstream mediator of TGF-β1 responses [26], [27], although some studies suggest that CTGF has a profibrotic effect independent of TGF-β1 [28], [29]. Lasky et al. reported an increase in CTGF mRNA expression in both human and murine lung fibroblasts stimulated with TGF-β *in vitro*, and CTGF mRNA expression was up-regulated in bleomycin-induced lung fibrosis in mice *in vivo* [30]. Our analysis of BALF showed a reduction in CTGF production without a decrease in TGF-β concentration in S1P3-deficient mice in which lung fibrosis was attenuated. This reduction of fibrosis in S1P3 KO mice may be due to a decrease in the number of total cells followed by a reduction in CTGF concentration in BALF; however, it cannot explain fully the dissociation of CTGF and TGF-β concentrations in BALF.

Several *in vitro* reports suggest that cross-talk occurs between S1P and TGF-β signaling. Xin et al. reported that S1P transactivated the TGF-β receptor and triggered activation of Smads followed by CTGF gene transcription in renal mesangial cells [31]. Cencetti et al. showed that TGF-β1 up-regulated sphingosine kinase-1 in C2C12 myoblasts in a Smad-dependent manner and concomitantly induced high levels of S1P3 expression [32]. They also reported that inhibition of S1P3 strongly attenuated the profibrotic response to TGF-β1 [32]. Lowe et al. demonstrated that TGF-β-stimulated collagen production in cardiac fibroblasts involves S1P signaling, whereby intracellular S1P produced by SphK1 is released and acts in an autocrine/paracrine fashion to increase collagen production [33]. Milara et al. reported that transformation of alveolar type II cells to mesenchymal cells was induced via S1P2 and S1P3 activation. They showed that S1P3 directly increased the phosphorylation of Smad3; by contrast, an S1P2 antagonist indirectly suppressed phospho-Smad3 expression by reducing TGF-1β secretion [20]. These reports suggest that S1P3 receptor signaling plays an important role in fibrosis, and our results indicate that this signaling acts via CTGF expression. On the other hand, Sobel et al. reported that S1P mediates ECM synthesis by normal human lung fibroblast-derived myofibroblasts via S1P3 and S1P2 receptors using Smad-independent pathways, in contrast to TGF-β1, which activates Smad2/3 signaling [34]. They showed that S1P activates PI3K/Akt and ERK1/2 signaling to induce ECM synthesis, suggesting the presence of another mechanism in the S1P3-CTGF axis.

In this study, we clarified the importance of S1P3 receptor signaling on bleomycin-induced pulmonary inflammation and fibrosis. Our findings suggest that this signaling occurs via CTGF expression, resulting in the onset of pulmonary fibrosis. This pathway could be a therapeutic target for pulmonary diseases such as IPF or interstitial pneumonia, although further investigation is required to elucidate the role of S1P3 signaling in these diseases.

## Supporting Information

Figure S1 **Survival rate after administration of bleomycin.** The survival rate after administration of bleomycin. S1P3 knockout (KO) mice (n = 31) and wild-type (WT) mice (n = 34) received a single intratracheal dose of bleomycin (2.15 U/kg). Data from four independent experiments were combined; n = 4–10 mice/group in total. Survival rate of WT mice decreased to 61.8% (21/34) on the eleventh day and survival of S1P3 KO mice to 87.1% (27/31). The survival rate of S1P3 KO mice after administration of bleomycin was significantly higher than that of WT mice (p = 0.039; the data were analyzed by log-rank test).
(TIF)
