# Supplementary figures and images for: Knock Out of S1P3 Receptor Signaling Attenuates Inflammation and Fibrosis in Bleomycin-Induced Lung Injury Mice Model
Source: PLoS One. 2014 Sep 8;9(9):e106792. doi: 10.1371/journal.pone.0106792 (PMC4157792; doi:10.1371/journal.pone.0106792)

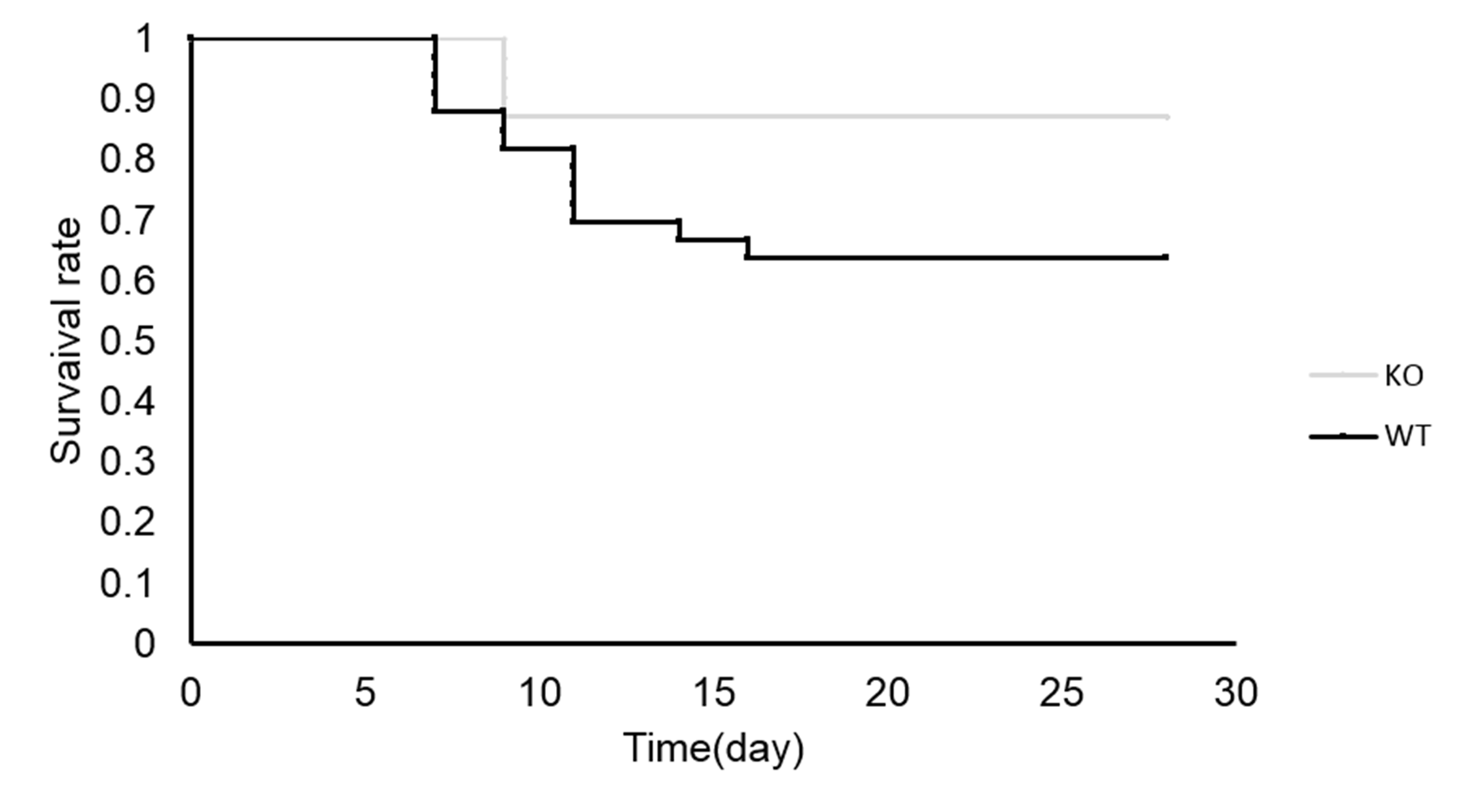

Supplement: Figure S1 — Survival rate after administration of bleomycin. The survival rate after administration of bleomycin. S1P3 knockout (KO) mice (n = 31) and wild-type (WT) mice (n = 34) received a single intratracheal dose of bleomycin (2.15 U/kg). Data from four independent experiments were combined; n = 4–10 mice/group in total. Survival rate of WT mice decreased to 61.8% (21/34) on the eleventh day and survival of S1P3 KO mice to 87.1% (27/31). The survival rate of S1P3 KO mice after administration of bleomycin was significantly higher than that of WT mice (p = 0.039; the data were analyzed by log-rank test). (TIF) [file pone.0106792.s001.tif]
